# 4511 Bio-Compatible Implantable Oxygen Sensor Technology with Real-Time Monitoring of Surgical Flaps and Reimplantation

**DOI:** 10.1017/cts.2020.292

**Published:** 2020-07-29

**Authors:** Preet Patel, Mohamed Ibrahim, Bruce Klitzman

**Affiliations:** 1 Duke University; 2 Dept of Plastic and Reconstructive Surgery, Duke University

## Abstract

OBJECTIVES/GOALS: Current surgical flap and replantation monitoring techniques have limitations in detecting the pathologic state, calibration and cost-to-patient issues. Our hypothesis is that novel implantable oxygen sensors can provide a more efficient, accurate, and reliable monitoring of tissue oxygenation. METHODS/STUDY POPULATION: Experimental sensors were used with an exogenous remote used as a reader once implanted (Fig. 1) A rat tissue perfusion model with three regions of an SIEA flap as well as into adjacent control sites was made (Tip, Middle, and Base) Blood flow was greatest at the base, diminishing towards the Tip, thus creating a perfusion gradient. Changes in tissue oxygen tension PO2 were estimated by the steady-state fluorescence of the optical sensors using an IVIS imaging system. The sensors were used to collect data from days 0, 3, and 7 as a reading of Tissue Oxygen Tension (TOT) with ANOVA used to assess for statistical significance in blood oxygen data with respect to relative perfusion status. RESULTS/ANTICIPATED RESULTS: Inspired FiO2 was decreased from 100% to 12% with a corresponding change in the TOT readings from all sensors. (Fig. 2) The tip portion of the flap demonstrated the most profound detection of tissue necrosis, with the middle demonstrating the second most necrosis and the base demonstrating the least with correlating TOT sensor readings. (Fig. 3) Acute vascular compromise of the feeding blood vessels in the pedicle was immediately detected within 70 seconds (*p<0.05). (Fig. 4) DISCUSSION/SIGNIFICANCE OF IMPACT: This study introduces and validates a recent technique to monitor acute vascular occlusion, flap viability, and necrosis in the immediate postoperative period in a validated rodent model. Future directions of this novel technology will aim to reproduce these findings in clinical feasibility studies.

